# Macroalgae as a Valuable Source of Naturally Occurring Bioactive Compounds for the Treatment of Alzheimer’s Disease

**DOI:** 10.3390/md17110609

**Published:** 2019-10-25

**Authors:** Tosin A. Olasehinde, Ademola O. Olaniran, Anthony I. Okoh

**Affiliations:** 1 Applied Environmental and Microbiology Research Group (AEMREG), Department of Biochemistry and Microbiology, University of Fort Hare, Eastern Cape, Alice 5700, South Africa; aokoh@ufh.ac.za; 2 SAMRC Microbial Water Quality Monitoring Centre, University of Fort Hare, Eastern Cape, Alice 5700, South Africa; 3 Nutrition and Toxicology Division, Food Technology Department, Federal Institute of Industrial Research, Oshodi, Lagos PMB 21023, Nigeria; 4 Discipline of Microbiology, School of Life Sciences, College of Agricultural, Engineering and Science, University of Kwazulu-Natal, Durban 4001, South Africa; Olanirana@ukzn.ac.za

**Keywords:** Alzheimer’s disease, seaweeds, cholinesterases, beta-secretase, beta-amyloid aggregation, neuroprotection

## Abstract

Alzheimer’s disease (AD) is a neurological condition that affects mostly aged individuals. Evidence suggests that pathological mechanisms involved in the development of AD are associated with cholinergic deficit, glutamate excitotoxicity, beta-amyloid aggregation, tau phosphorylation, neuro-inflammation, and oxidative damage to neurons. Currently there is no cure for AD; however, synthetic therapies have been developed to effectively manage some of the symptoms at the early stage of the disease. Natural products from plants and marine organisms have been identified as important sources of bioactive compounds with neuroprotective potentials and less adverse effects compared to synthetic agents. Seaweeds contain several kinds of secondary metabolites such as phlorotannins, carotenoids, sterols, fucoidans, and poly unsaturated fatty acids. However, their neuroprotective effects and mechanisms of action have not been fully explored. This review discusses recent investigations and/or updates on interactions of bioactive compounds from seaweeds with biomarkers involved in the pathogenesis of AD using reports in electronic databases such as Web of science, Scopus, PubMed, Science direct, Scifinder, Taylor and Francis, Wiley, Springer, and Google scholar between 2015 and 2019. Phlorotannins, fucoidans, sterols, and carotenoids showed strong neuroprotective potentials in different experimental models. However, there are no data from human studies and/or clinical trials.

## 1. Introduction

Macroalgae, also known as seaweeds, are marine organisms and reservoirs of natural biologically active compounds. Different classes of macroalgae include rhodophyta (red algae), chlorophyta (green algae), and phaeophyta (brown algae). There are over 4000 red, 900 green, and 1500 brown species of macroalgae all over the world [1]. Most of the brown macroalgae are able to thrive in temperate and cool waters while green and red algal species exist in tropical and subtropical waters [1]. Several species of macroalgae thrive in their habitat at extreme conditions due to their capacity to develop defense mechanism via the release of some secondary metabolites. Historically, seaweeds have been used traditionally, especially in Asian countries, as herbal medicine for the treatment of tumors, urinary disease, gastrointestinal problems, cough, boils, hemorrhoid, ulcers, asthma, and headaches [2]. The appreciable levels of iodine in some edible seaweeds also make them a good choice for the treatment of goiter [3,4]. Specifically, species such as *Ulva* spp., *Laminaria japonica*, *Porphyra* spp., and *Sargassum fusiforme* have been used for the treatment of scrofula (cervical tuberculosis), edema, and goiter [3]. Furthermore, a combination of *Ecklonia* spp. and *Sargassum* spp. are used as herbs in Chinese medicine for the treatment of tumors, liver cirrhosis, and spleen enlargement [5]. Seaweeds are commonly consumed locally as vegetables and in salad. Some species of macroalgae are consumed as part of a staple diet because they are rich in functionally active compounds such as phenolic compounds, alkaloids, sterols, omega-6 fatty acids, antioxidants, carotenoids, and phenolic compounds [6]. They are also used as ingredients for dietary supplements, nutraceuticals, and pharmaceuticals. Some of the applications of some macroalgal species can be seen in their use as ingredients for the production of flavor, meat, cereal, and dairy products [7]. Much attention has been on marine macroalgae for the development of new drugs, nutraceuticals, and dietary supplements due to their beneficial effects as antioxidant [8], anti-tumor [9], anti-inflammatory [10], antidiabetic [11], anti-hypertensive [12], and antibacterial [13] agents. Evidence has shown that macroalgal-derived compounds are capable of improving learning and memory function in neurodegenerative conditions [14]. The neuroprotective effects of biologically active compounds from some macroalgae against neurodegenerative diseases has been described by Alghazwi et al. [15].

Alzheimer’s disease (AD) is the most common form of dementia and has become a major health problem among aged individuals [16]. AD is characterized by cholinergic dysfunction, cognitive impairment, memory loss, neuronal death, and behavioral disturbances. The pathogenesis of AD involves complex mechanisms and impairment of the neurological cascade involved in memory function. The early onset of this disease has been diagnosed in persons less than 65 years. However, more than 90% of cases diagnosed are associated with the late onset of AD and this occurs mostly in individuals above 65 years of age [17]. The development of early onset AD has been linked with genetic mutations, especially genes that are responsible for Aβ peptide production (amyloid precursor protein (APP gene)), preselinin 1 (PS1), and preselinin 2 (PS2) genes [18]. Evidence has shown that dysregulation in the expression of these genes may account for about 5–10% of diagnosed cases of early onset AD [17,18]. Furthermore, apolipoprotein E (APO-E) polymorphic alleles has been identified as a major genetic risk factor for the development of early onset and late onset AD [19]. APOε4 alleles have been shown to trigger cognitive decline and cerebral amyloid angiopathy in aged individuals [20]. APOE is produced in astrocytes in the central nervous system, and it is important in the regulation of lipid homeostasis and beta-amyloid (Aβ) metabolism. It also contributes to the formation of Aβ plaques and development of cerebral amyloid angiopathy [21]. Furthermore, APOε4 has also been linked to tau pathology [22]. The molecular mechanisms involved in the development of sporadic late onset of AD is not well known; however, previous reports have shown that it is linked to oxidative stress [23], loss of cholinergic signaling [24], accumulation of Aβ plaques [25], and formation of neurofibrillary tangles [26]. Moreover, a recent report has shown that hyperphosphorylation of tau proteins is a major causative factor involved in the development and progression of AD [27]. Hence, recent research investigations are considering tau pathology as a therapeutic strategy for the management of AD. 

Previous experimental investigations have established that natural products could be effective in the management of Alzheimer’s disease and have been suggested as an alternative therapeutic approach compared to synthetic agents [28,29,30]. The discovery of novel natural compounds from different species of marine macroalgae represents an important source of biologically active compounds with strong neuroprotective potentials. This present review report provides current knowledge on neuroprotective potentials of macroalgal-derived natural compounds and their effects on pathological mechanisms (oxidative stress-induced neurodegeneration, cholinergic dysfunction, and beta-secretase activities, as well as glutamate and metal-induced neurotoxicity and beta-amyloid aggregation) linked to Alzheimer’s disease.

## 2. Methods

A literature search was done in different databases including Web of Science, Scopus, Scifinder, PubMed, Google scholar, Science direct, Springer, Taylor and Francis, and Wiley to identify reports published between 2015 and 2019 that are related to antioxidant activity and neuroprotective effects of macroalgal extracts and compounds as well as their modulatory effects on biomarkers linked with Alzheimer’s disease. Articles revealing reports on the neuroprotective properties and modulatory effects of macroalgal extracts on biomolecules related to other neurodegenerative diseases such as Parkinson’s disease, Huntington’s disease, ischemia, and stroke were not considered in this study.

## 3. Etiology of AD

**Figure 1:**
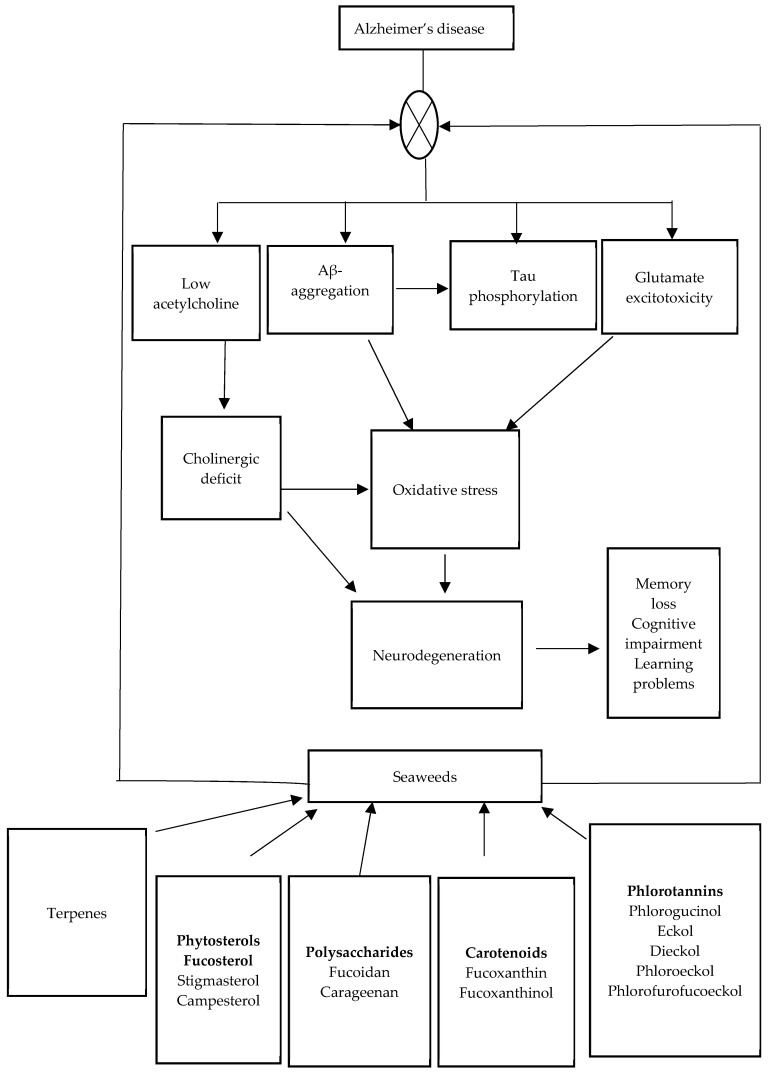
Mechanism of action of bioactive compounds from macroalgae against Alzheimer’s disease.

The occurrence of AD amongst aged individuals has been estimated to increase annually due to the complexity of its pathological mechanisms [31]. The etiology of AD is not well understood due to the multifactorial mechanisms underlying the disease process. Some of the factors that have been linked to the development and progression of AD include ageing, cholinergic deficit, Aβ aggregation, tau hyperphosphorylation, oxidative stress, neuro-inflammation, and diabetes. Figure 1 shows a complex cascade and set of mechanisms involving the development of AD. The cure for AD has not been discovered due to the complexity of the neuropathological mechanisms involved in the development and progression of the disease [32]. Fewer achievements have been attained in terms of effective treatment of the disease; however, much focus has been on early detection and prevention. Adequate diagnostic methods for AD is one of the challenging factors that has been encountered due to non-availability of reliable biomarkers. The current therapeutic approach used for the management of AD involves the use of cholinesterase inhibitors and the N-methyl-d-aspartate receptor antagonist. These drugs do not have the capacity to halt the progression of the disease. Effective therapeutic agents or disease modifying drugs that are capable of stopping or preventing the clinical symptoms of AD are currently under extensive investigation [33,34]. However, seaweed-derived compounds have shown great potential as an alternative strategy for the management of AD (Figure 1).

## 4. Therapeutic Role of Some Macroalgae in the Management of AD

### 4.1. Evidence from In Vitro Studies

Some in vitro models have been used to determine the neuroprotective potentials of some macroalgal species against biomarkers of AD. Extracts derived from several species of macroalgae have been tested for their cholinesterase and beta-secretase (BACE-1) inhibitory activities. Neuronal cells have also been used as experimental models to determine the neuroprotective activities of macroalgal-derived compounds and extracts.

#### 4.1.1. Cholinesterase Inhibitory Activity

Acetylcholinesterase (AChE) and butyrylcholinesterase (BChE) are important enzymes involved in the regulation of acetylcholine (ACh) in the synaptic cleft of neurons to promote cognitive function [35,36]. However, loss or rapid degradation of acetylcholine leads to cholinergic dysfunction and ultimately memory impairment [37,38]. Hence, cholinesterases have been developed to alleviate cholinergic deficit by restoring ACh levels and improving cognitive function [39,40]. Seaweed-derived biologically active compounds have been reported to exhibit inhibitory effects on enzymes associated with Alzheimer’s disease (Table 1). Results from some in vitro studies from our laboratory revealed that aqueous-ethanol extracts rich in phlorotannins, phenolic acids, and flavonoids from *Ecklonia maxima*, *Gelidium pristoides*, *Gracilaria gracilis*, and *Ulva lactuca* exhibit acetylcholinesterase and butyrylcholinesterase inhibitory activities [41]. Furthermore, sulfated polysaccharides obtained from *Ulva rigida* as well as the aforementioned algal species also showed potent inhibitory effects on BChE and AChE in vitro [42,43]. Purified fractions of *Gelidiella acerosa* showed AChE and BChE inhibitory activity [44]. Phytol was identified in the fraction as the most effective constituent. In the same study, molecular docking analysis revealed that phytol tightly binds to the arginine residue at the active site of the enzyme, thereby changing its conformation and exerting its inhibitory effect. Rengasamy et al. [45] reported AChE inhibitory activity of *Codium duthieae*, *Amphiroa beauvoisii*, *Gelidium foliaceum*, *Laurencia complanata*, and *Rhodomelopsis africana*. *Hypnea musciformis* and *Ochtodes secundiramea* extracts showed weak inhibitory activity (less than 30% inhibition) on AChE. Jung et al. [46] also reported AChE and BChE inhibitory effects of methanol extracts of *Ecklonia cava*, *Ecklonia kurome*, and *Myelophycus simplex*. Glycoprotein isolated from *Undaria pinnatifida* showed dose responsive inhibitory effects on butyrylcholinesterase and acetylcholinesterase activities [47]. 

The IC_50_ values revealed that the glycoprotein exhibited higher inhibitory effect on AChE (63.56 µg/mL) than BChE (99.03 µg/mL). The enzyme inhibitory activities of the extracts were attributed to the presence of monoterpenes, which are reversible competitive inhibitors of the AChE. Shanmuganathan et al. [48] attributed the inhibitory effect of acetone extracts from *P. gymnospora* to alpha bisabolol.

In a recent study, fucosterol isolated from *Sargassum horridum* demonstrated potent inhibition against AChE activity [49]. Kinetic studies revealed that fucosterol showed competitive and non-competitive inhibition due to its high binding affinity to AChE compared to neostigmine. Choi et al. [50] reported that phlorofucofuroeckol isolated from *Ecklonia cava* exhibited potent inhibitory effects against BChE with an activity of over 100 fold higher than AChE inhibition.

#### 4.1.2. BACE-1 Inhibitory Activity

BACE-1 has been identified as one of the prime therapeutic targets for the treatment of AD. It is a membrane-bound aspartyl protease that regulates Aβ production in the metabolism of amyloid precursor proteins. An increase in BACE-1 activity as well as elevated protein expression levels have been shown to trigger rapid production of Aβ protein and sporadic AD [51]. Cheng et al. [52] suggested that elevated levels of BACE-1 activity and increase in tumor necrosis factor (TNFa) expression may contribute to mild cognitive impairment and early events of AD. Furthermore, elevated BACE-1 activity also contributes to the increased number of plaques around the neurons and reduces cognitive ability of AD patients. High BACE-1 activity was also attributed to neurodegeneration and neurological decline in a transgenic mice model of AD [53]. The search for potent BACE-1 inhibitors has been a huge task as many inhibitors that have been developed have failed clinical tests. Some marine algal species have shown good potential as BACE-1 inhibitors (Table 1). Findings from our laboratory revealed that aqueous-ethanol extracts of *G. pristoides*, *E. maxima*, *U. lactuca*, and *G. gracilis* containing phlorotannins, flavonoids, and phenolic acids exhibited strong BACE-1 inhibitory activity with percentage inhibition of 97.2, 83.3, 86.9, and 91.2% at the highest concentration (120 µg/mL) [41]. In another study, fucoidan, ulvan, and carrageenan obtained from *E. maxima*, *U. lactuca*, and *G. pristoides* also inhibited BACE-1 activity with percentage inhibition of 87.1, 71.2, and 51.3%, respectively, at the highest concentration (5.0 mg/mL) [43]. Jung et al. [54] also reported the potency of fucosterol and fucoxanthin isolated from *Undaria pinnatifida* and *Ecklonia stolonifera*, respectively, against BACE-1 activity. Fucoxanthin and fucosterol showed mixed and non-competitive types of inhibition, respectively, and their inhibitory activities were attributed to strong binding to hydroxyl groups of specific amino acid residues at the active site of the enzyme. Seong et al. [55] elucidated that monoterpenoids obtained from *S. sagamianum* exhibited potent BACE-1 inhibitory activity in vitro. The isolated compounds, saraquinoic and sargahydroquinoic acids, as well as sargachromenol interacted with the catalytic aspartyl residues and allosteric sites, thereby initiating tight binding to the enzyme, hence reducing its activity. Phlorofucofuroeckol isolated from *E. cava* also reduced BACE-1 activity [50]. Rafiquzzaman et al. [47] isolated and purified glycoproteins from *Undaria pinnatifida* and investigated their inhibitory effects on BACE-1 activity. The glycoprotein exhibited a dose dependent inhibitory effect on BACE-1. An insilico investigation on BACE-1 inhibitory potentials of glycyrrhizin and its metabolites isolated from *Hizikia fusiformis* revealed that 18α-glycyrrhetinic acid and 18β-glycyrrhetinic acid showed inhibitory effects against BACE-1 activity [56]. Moreover, 18β-glycyrrhetinic acid showed two-fold potent inhibitory activity compared with quercetin. The inhibitory activity of these compounds were attributed to their strong capacity to bind to the amino acid residues at the active site of BACE-1 via hydrogen bonds.

#### 4.1.3. Action against Glutamate-Induced Neurotoxicity in Neuronal Cells

Glutamate is an important neurotransmitter responsible for memory, learning, and cognitive function. However, excess levels of glutamate activate NMDA receptors and trigger the production of Aβ peptide. Previous studies have highlighted two major pathways that trigger glutamate excitotoxicity; these include disruption of calcium homeostasis, which leads to the production of reactive oxygen species and neuronal death, as well as alterations in cysteine uptake due to high levels of glutamate [57]. This leads to imbalance of cystine homeostasis, limited levels of glutathione, and rapid production of reactive oxygen species. Hence, biologically active compounds capable of protecting the brain cells against glutamate excitotoxicity may be a good therapeutic intervention. Macroalgae are good sources of compound with the capacity to attenuate glutamate excitotoxicity in neuronal cells. Acetone extracts from two edible seaweeds (*Saccahrina latissima* and *Fucus serratus*) improved cell viability in glutamate-induced neurotoxicity in SH-SY5Y cells [58]. Phlorofucofuroeckol isolated from *E. cava* protected neurons against cell death, improved mitochondrial dysfunction, and regulated intracellular production of reactive oxygen species (ROS) in PC12 cells [59].

#### 4.1.4. Protection against Aβ-Induced Neurotoxicity

Accumulation of Aβ peptide is one of the hallmarks of AD pathology. Aβ is a pathogenic peptide released from amyloid precursor protein, which aggregates to form toxic plaques around the neurons [60]. Currently, no drug has been developed to combat Aβ aggregation and its pathological processes in AD. Some species of macroalgae have been identified as potential sources of compounds capable of attenuating Aβ-induced neurotoxicity in AD models (Table 2).

Crude extracts of some algal species have been reported to inhibit amyloid formation and cause dis-aggregation of matured beta-amyloid fibrils [48,61,62]. In a study carried out by Alghazwi et al. [63], some species of Australian brown, green, and red algae attenuated Aβ-induced toxicity in PC12 cells. Fucosterol from *Padina australis* was evaluated for its neuroprotective effects in SH-SY5Y cells treated with Aβ [64]. The result revealed that fucosterol ameliorated the neurotoxic effect of Aβ and triggered the downregulation of APP expression. Alghazwi et al. [65] also reported the neuroprotective effects of fucoidans isolated from *Undaria pinnatifida* and *Fucus vesiculosus* via their inhibitory effect on Aβ aggregation and Aβ_1–42_-induced cytotoxicity in PC12 cells. In the same study, phlorotannins such as 7-phloroeckol, phlorofucofuroeckol, and dieckol also protected PC-12 cells against Aβ-induced neurotoxicity, reduced ROS production, and restored intracellular levels of Ca^2+^. However, dieckol exhibited moderately weak neuroprotective effects compared to 7-phloroeckol and phlorofucofuroeckol. Furthermore, phloroglucinol isolated from *E. cava* reduced ROS generation caused by Aβ-induced neurotoxicity in HT-22 cells [66]. Another unique phlorotannin (eckmaxol) isolated from *E. maxima*, also exhibited anti-amyloidogenic activity [67] (Table 2). Furthermore, fucoxanthin and fucosterol also attenuated amyloid oligomer-induced neurotoxicity in neuronal cell line models [68,69,70]. Wei et al. [71] also showed that fucoidan inhibited apoptosis in PC12 cells via activation of caspases, prevention of cytochrome c release, and upregulation of X-linked inhibitor of apoptosis (XIAP) in Aβ-induced PC-12 cells.

#### 4.1.5. Antioxidant Activity of Macroalgae and AD

**Figure 2:**
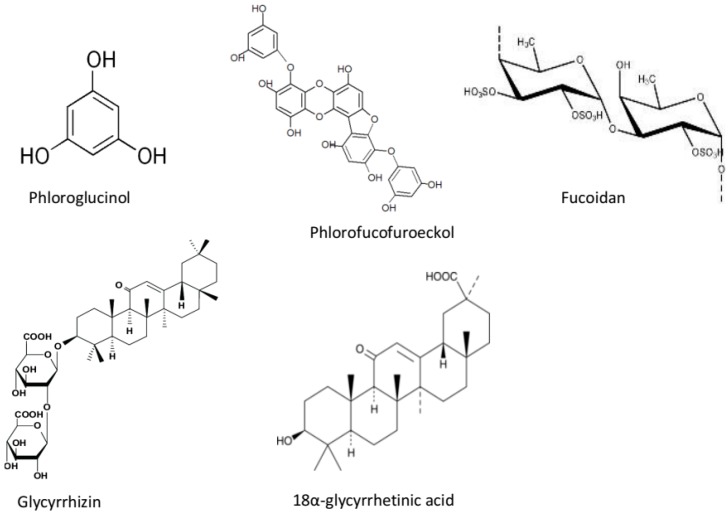

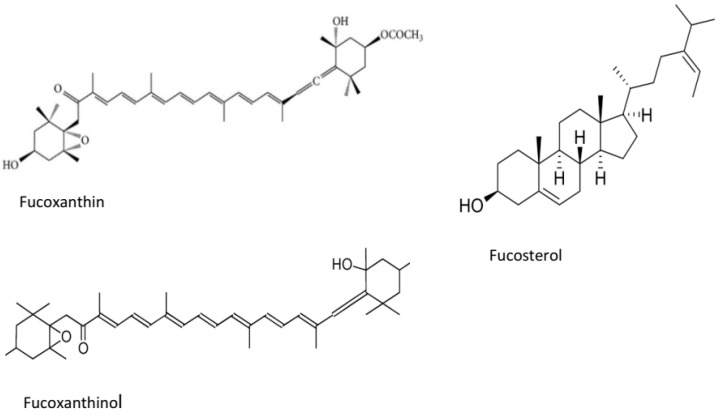
Chemical structures of some neuroprotective compounds in marine seaweeds.

Antioxidants have been identified as an effective therapeutic strategy for the delay of the progression of AD. This is due to the fact that elevated levels of ROS in the brain are associated with the progression of AD. Brain cells are highly susceptible to free radical attack due to high consumption of oxygen and lipid content as well as low antioxidant defense system. Hence, high levels of reactive oxygen species in brain cells may lead to lipid peroxidation, neurodegeneration, and ultimately cell death. Some species of macroalgae have been reported to exhibit neuroprotective effects via their antioxidant activities. Alghazwi et al. [15] reported the antioxidant activity of 49 compounds isolated from some brown, red, and green macroalgal species. Most of these compounds were identified as polysaccharides, phlorotannins, and terpenoids. Recent findings on the antioxidant activity of macroalgae revealed that some other algal species exhibit radical scavenging and metal chelating activities in vitro (Table 3 and Figure 2). Sathya et al. [72] reported the DPPH radical scavenging activity of methanol, ethylacetate, and dichloromethane extracts of *Cystoseira trinodis* with scavenging activity of 50%, 54%, and 69% respectively. The ethyl acetate and butanol fractions of *Sargassum fusiforme* were also reported to exhibit radical scavenging activity [73]. Methanol extracts of some *Gracilaria* spp., *Lesonia* spp., *Laminaria japonica*, and *Ascophyllum nodosum* also exhibited DPPH and ABTS radical scavenging activities and ferric reducing properties [74,75]. Furthermore, acetone extracts from *Ulva lactuca* and *Entermorpha intestinalis* [76], and ethanol and hexane extracts from *Pterocladiella capillacea* and *Osmindaria obtusiloba* [77] including aqueous extracts from *Ascophyllum nodosum*, *Bifurcaria bifurcate*, and *Fucus vesiculosus* [78] also exhibited potent antioxidant activity via their radical scavenging activities, ferric reducing properties, and inhibition of lipid oxidation. The antioxidant activities of these algal extracts were attributed to the presence of phlorotannins. Pinteus et al. [79] reported the antioxidant activity of the methanol and dichloromethane extracts of 27 red, green, and brown macroalgal species through their oxygen radical antioxidant capacity and DPPH radical scavenging activity. The study showed that the methanol extracts of the brown algal species exhibited the highest antioxidant activities. Similarly, out of the hexane, ethyl acetate, and methanol extracts of seven algal species reported by Chiboub et al. [80], only *Cytoseira sedoides* (hexane, ethyl acetate, and methanol), *Padina pavonica* (ethyl acetate extract), *Cladostephus spongiosum* (ethylacetate and methanol), and *Halopteris scoparia* (methanol extract) exhibited DPPH and ABTS radical scavenging activity above 50%. 

Crude fucoidans extracted from *Sargassum* sp. also exhibited antioxidant activity via their ferric reducing antioxidant properties and hydroxyl radical scavenging activities [81]. Purified fractions of *H. elongata* and *Macrocytis pyrifera* extracts exhibited antioxidant activity and this effect was attributed to the presence of phenolic terpenes, flavonoid derivatives, and phlorotannins [82,83]. Phenolic rich extracts from *E. maxima*, *U. lactuca*, *U. rigida*, *G. gracilis*, and *G. pristoides* also attenuated Zn-induced neurotoxicity and protected hippocampal neuronal cells (HT-22) against neuronal damage via inhibition of apoptosis, reduction of nitric oxide and malondialdehyde production, and improvement of antioxidant status [84,85]. The neuroprotective effects of the phenolic extracts could be linked to the radical scavenging and metal chelating activities of some of the bioactive constituents, which include phloroglucinol, ferulic acid, dihydroxybenzoic acid, 3,7-dimethyl quercetin, 5,7-dimethoxyflavone, dihydronaringenin-O-sulfate, apigenin, 7,2,4-trihydroxyisoflavanol, and kaempferol 3-(6-acetyl galactoside)7-rhamnose [41,84,85]. Polysaccharides such as fucoidans, alginates isolated from *Sargassum* spp., *Laminaria japonica*, *Cystoseira trinodi,* and *Nizimuddinia zanardini* [86,87,88,89,90,91], as well as protein extracts obtained from *Ulva* spp. and *Gracilaria* spp. [92] also showed potent radical scavenging activities. Furthermore, the report of Mohibbullah et al. [93] revealed that *Porphyra yezoensis* (an edible red alga) extracts induced synaptogenesis, increased neuronal survivability, and prevented neuronal death due to its radical scavenging ability. The neuroprotective effect of the extracts was attributed to taurine, an active component that improved neuronal development and maturation. Some studies also showed that carotenoids such as fucoxanthin and fucoxanthinol isolated from brown alga *Himanthalia elongata* [94], *Sargassum horneri* [95], and *Undaria pinnatifida* [96] exhibit neuroprotection via their antioxidant activity, as revealed by their radical scavenging activities, inhibition of apoptosis, intracellular reactive oxygen species and malondialdehyde production, attenuation of mitochondria membrane dysfunction, and DNA fragmentation. Glycoprotein isolated from *U. pinnatifida* increased superoxide dismutase and xanthine oxidase activities in hippocampal neuronal cells, which suggest its ability to prevent neurodegeneration [47]. Table 3 shows recent findings on the antioxidant activities of different classes of algal extracts and compounds. Antioxidant activity has been linked with neuroprotective effects due to the ability of antioxidants to suppress neurodegeneration, prevent neuronal death, and halt the progression of AD. Hence macroalgae are good sources of antioxidants with neuroprotective effects.

### 4.2. Evidence from In Vivo Studies

Neuroprotective effects of macroalgal extracts and compounds have been determined using different targets, which include oxidative stress, cholinergic function, beta-amyloid aggregation, apoptosis, and behavioral studies in in vivo models.

#### 4.2.1. Neuroprotective Activities of Some Macroalgal Extracts

Crude extracts of some macroalgal species have shown neuroprotective potentials against some markers of Alzheimer’s disease in vivo. Syad and Devi [97] confirmed that benzene extracts of *G. acerosa* attenuated neurotoxicity induced by AB_25–35_ in Swedish mice brain. *G. acerosa* improved memory function by attenuating cholinergic dysfunction via inhibition of acetylcholinesterase and butyrylcholinesterase. The extracts also inhibited BACE-1 activity in vivo, hence suppressing Aβ neurotoxicity. In the same study, *G. acerosa* extract protected mouse brain against lipid peroxidation and reduced caspase 3-activity and Bax expression, which suggest its effect against neuronal death. Acute and sub-acute toxicity experiments carried out on the benzene extract did not show any toxic effects on different organs in Swedish mice. Furthermore, a phlorotannin-rich fraction of *Ishige foliacea*, an edible brown seaweed, was reported to improve memory function in scopolamine-induced rat brain via reduction of lipid peroxidation, increase in superoxide dismutase activity and glutathione levels, as well as upregulation of brain-derived neurotrophic factors (BNDF), cyclic-AMP response binding protein (CREB), and phosphorylated extracellular signal regulated kinase (ERK) [98]. Choi et al. [99] found that ethanol extracts of *U. pinnatifida* improved cognitive dysfunction in mouse brain. The results of the study indicate that treatment with ethanol extracts of *U. pinnatifida* caused repairing effects in memory and restored spine density and morphology via increase in latency time in the passive avoidance test and dendritic spine in hippocampal neurons of scopolamine-induced rats. Similarly, fucoidan enhanced spatial learning and memory, which was impaired by infusion of D-galactose in mice brain [71]. In the same study, D-galactose reduced acetylcholine levels and choline acetyltransferase activity while acetylcholinesterase activity was significantly high, which suggests cholinergic dysfunction. However, treatment with fucoidan (50 mg/kg) reversed the levels of acetylcholine as well as choline acetyltransferase and acetylcholinesterase activities, which indicates an improvement in cholinergic function. Similarly, the neuroprotective effect of a fucoidan (SFPS65) isolated from *Sargassum fusiforme* was found in scopolamine and sodium nitrate-induced rats investigated by Hu et al. [100]. The authors suggested that SFPS65A improved stepdown latency, which was disrupted by scopolamine, and mitigated shock number and total wrong frequency. SFPS65A also attenuated spatial learning and memory dysfunction caused by ethanol and sodium in mice brain. 

#### 4.2.2. Neuroprotective Effects of Macroalgal-Derived Compounds

Several compounds have been identified and isolated from macroalgae; however, only few have been tested against biomarkers of AD in vivo. Fucoidan present brown alga showed neuroprotective Aβ-induced neurotoxicity in a transgenic *Caenorhabditis elegans* AD model [101]. The result of the study revealed that fucoidan reduced Aβ accumulation by improving proteolysis and attenuation of Aβ-induced ROS production Oh et al. [68] investigated the effect of fucosterol isolated from *E. stolonifera* on Aβ-induced cognitive dysfunction, again in rats. The study showed that fucosterol may enhance cognitive function in aging-induced endoplasmic reticulum stress and memory impairment. Fucosterol attenuated Aβ-induced cognitive dysfunction via upregulation of BDNF–TRkB–ERK1/2 expression in rats’ dentate gyrus. Lin et al. [102] evaluated the effect of fucoxanthin in scopolamine-induced memory impairment in rats’ brains. Fucoxanthin reversed scopolamine-induced memory dysfunction via inhibition of acetylcholinesterase, and decrease in choline acetyltransferase activity and BDNF expression. Structure–activity analysis showed that fucoxanthin binds to the peripheral anionic site of AChE, hence decreasing the activity of the enzyme. Similarly, the study of Xiang et al. [103] revealed that fucoxanthin attenuated Aβ-induced cognitive impairment in mice brain. The inhibitory effects of fucoxanthin against Aβ oligomerization and aggregation was attributed to its hydrophobic interaction with the peptide, hence preventing conformational transition and self-aggregation of the Aβ peptide. Fucoxanthin also improved spatial learning and memory via the water maze tests and reversed the low levels of glutathione and superoxide dismutase and high levels of malondialdehyde in Aβ-induced mice. Furthermore, fucoxanthin activated the nuclear factor eythroid 2-related factor (NrF-2)-antioxidant response element (ARE) pathway and reversed upregulation of malondialdehyde and glutathione peroxidase activity in a rat model of traumatic brain injury, hence alleviating neurological deficits, neuronal apoptosis, brain lesion, and cerebral edema [104]. Yang et al. [66] reported that phloroglucinol isolated from *E. cava* attenuated cognitive deficit in mice brain. The study revealed that phloroglucinol may delay the onset or progression of AD due to its protective effects against the decrease in dendritic spine density, synaptophysin, and post synaptic density protein 95 (PSD-95). Yang et al. [105] also confirmed that oral administration of phloroglucinol improved impaired cognitive function in 5XFAD mice via reduction of 4-hydroxylnonenal, Aβ plaques, and pro-inflammatory cytokine production, as well as attenuation of glial reactivation. 

## 5. Conclusions

This review provides evidence that macroalgae exhibit neuroprotective effects and could be important sources of biologically active compounds with therapeutic potential for the management of AD. Despite the identification of several macroalgal species and their various biological activities, only few have been explored for their neuroprotective effects against pathological mechanisms involved in Alzheimer’s disease. The neuroprotective activities of some macroalgal-derived compounds and extracts via attenuation of cholinergic deficit, Aβ aggregation, oxidative damage to neurons, and glutamate excitation, which have been established in recent findings, could be a significant approach for the management and treatment of AD. However, further investigations are needed to explore other species. Macroalgal extracts with potential neuroprotective activities should be characterized and purified, and their active constituents should be isolated. Further studies are also required to determine the mechanisms of action of macroalgal compounds and investigate their structure–activity relationship. Future research works on the neuroprotective effects of macroalgae may also focus on other targets linked with AD such as serotonin, somatostatin, tau hyperphosphorylation, neuro-inflammation, and metal-induced neurotoxicity, which have not been reported. Furthermore, most studies have shown in vitro neuroprotective effects of some algal species while in vivo experimental models are few. Further works should be done to determine the mechanism of action of macroalgal compounds in in vivo AD models. Compounds which have been established to exhibit neuroprotective effects in vivo, should be tested further in clinical trials.

## Figures and Tables

**Table 1:** Cholinesterase and beta-secretase inhibitory activities of macroalgal-derived extracts and isolated compounds.

Class of Compounds	Components	Algal Source	Mechanism of Action	Reference
Crude extracts	Benezene:ethyl acetate fraction	*G. acerosa*	Inhibition of AChE	[44]
	Methanol extracts	*E. cava*	Inhibition of AChE and BACE-1	
		*E. kurome*		
		*M. simples*	Inhibition of AChE	
Phlorotannins	Phlorofucofuroeckol	*E. cava*	Inhibition AChE, BChE, and BACE-1	[46]
Polysaccharides	Purified glycoprotein	*U. pinnatifida*	Inhibition of AChE, BChE and BACE-1``	[47]
Sterol	Fucosterol	*S. horridum*	Inhibition of AChE	[49]
Carotenoids	Fucoxanthin	*U. pinnatifida* *E. stolonifera*	Inhibition of BACE-1	[54]
Triterpenoid-saponin	Sarahydroquinoic acid	*S. serratifolium*	Inhibition of BACE-1	[55]
	Glycyrrhizin	*H. fusiformis*	Inhibition of AChE, BChE, and BACE-1	[56]
	18α-glycyrrhetinic acid			
	18β-glycyrrhetinic acid			

**Table 2:** Macroalgae extracts and compounds and inhibition of beta-amyloid-induced neurotoxicity.

Class of Compounds	Components	Algal Source	Mechanism of Action	Reference
Crude extracts	Aqueous extracts	*A. esculenta*	Inhibition of amyloid formation	[61]
	Acetone extracts	*P. gymnospora*	Anti-aggregation and dis-aggregation of amyloid fibrils	[48]
	Ether/benzene extracts	*G. acerosa*	Prevention of Aβ_25–35_ formation and dis-aggregation of pre-formed fibrils	[62]
Phlorotannins	Phloroglucinol	*E. cava*	Inhibition of Aβ-induced-cytotoxicity and protection against ROS accumulation in HT-22 cells	[64]
	Eckmaxol	*E. maxima*	Prevention of Aβ-induced neuronal apoptosis and decrease in intracellular ROS	[67]
Phytosterol	Fucosterol	*Padina australis*	Reduction of APP mRNA and inhibition of Aβ-induced neurotoxicity	[64]
		*E. stolonifera*	Attenuation of Aβ-induced cognitive dysfunction	[68]
Carotenoid	Fucoxanthin	*Sargassum horneri*	Attenuation of Aβ-oligomer-induced neurotoxicity in SYH-SY5Y cells	[69]
			Attenuation of Aβ-induced neurotoxicity in PC12-cells	[70]
Sulfated polysaccharides	Fucoidan	*U. pinnatifida* *F. vesiculosus*	Protection against Aβ_1–42_-induced neuronal death in PC-12 cells	[65]
			Inhibition of Aβ_25–35_-induced neurotoxicity in PC-12 cells	[71]

**Table 3:** Antioxidant activity of macroalgal-derived extracts and compounds.

Class of Compounds	Components	Algal Source	Mechanism of Action	Reference
Crude extracts	Methanol extract	*Ascophyllum nodosum*	ABTS and DPPH scavenging activity	[74]
		*Laminaria japonica*	Ferric reducing antioxidant property	
		*Lessonia trabeculate*		
		*Lessonia nigrescens*		
		*Gracilaria edulis*	DPPH, ABTS, and NO radical scavenging activities	[75]
		*Gracilaria corticata*		
		*Myelophycus simplex*	ABTS radical scavenging activity	[46]
		*Ecklonia cava*	Attenuation of H202-induced oxidative damage in SH-SY5Y cells	
		*E. kurome*		
	Acetone extract	*Ulva lactuca*	DPPH and superoxide anion scavenging activity	[76]
		*Entermorpha intestinalis*	Ferric reducing antioxidant property	
	Ethanol/hexane extract	*Pterocladiella capillacea*	DPPH radical scavenging activity	[77]
		*Osmindaria obtusiloba*	Metal chelating activity	
	Aqueous extract	*Ascophyllum nodosum*	DPPH, ABTS, and hydroxyl radical scavenging activities	[78]
		*Bifurcaria bifurcate*	Ferric reducing antioxidant capacity	
		*Fucus vesiculosus*	Inhibition of lipid oxidation	
Phlorotannins	Phlorotannin extract	*Macrocytis pyrifera*	DPPH radical scavenging activity	[83]
Polysaccharides	Fucoidan	*Sargassum glaucescens*	ABTS and DPPH scavenging and metal chelating activities	[85]
		*Sargassum polycystum*	DPPH radical scavenging activity Ferric reducing antioxidant property	[87]
	Fucoidan and alginate	*Cystoseira trinodis*	Ferric reducing antioxidant property	[88]
	Fucoidan and alginate	*Sargassum latifolium*	Hydroxyl radical scavenging activity	[89]
	Sodium alginate	*Nizimuddinia zanardini*	DPPH radical scavenging activity	[90]
	Polysaccharides	*Laminaria japonica*	DPPH and oxygen radical scavenging activity	[91]
	Fucoidan	*U. pinnatifida* *F. vesiculosus*	Attenuation of hydrogen peroxide-induced oxidative stress and apoptosis in PC-12 cells	[65]
			Activation of superoxide dismutase and glutathione in Aβ-induced neurotoxicity in PC-12 cells	[71]
Proteins	Protein extracts	*Ulva* spp.	Ferric reducing antioxidant property	[92]
		*Gracilaria* spp.	Oxygen radical absorption capacity	
Carotenoids	Fucoxanthin	*Himanthalia elongata*	Ferric reducing antioxidant property DPPH radical scavenging activity	[94]
		*Sargassum horneri*	Attenuation of H_2_O_2_-induced neuronal apoptosis and intracellular ROS	[95]
			Reduced malondialdehyde levels and SOD activity in Aβ-induced cell death in PC12 cells	[70]
	Fucoxanthinol	*Undaria pinnatifida*	Attenuation of oxidative stress in rats’ hippocampal neurons	[96]
